# Operative Hysteroscopy Versus Levonorgestrel-Releasing Intrauterine System (LNG-IUS) Insertion: A Prospective Observational Study on the Effect of Surgical and Medical Treatments on Sexual Functions in Women With Abnormal Uterine Bleeding

**DOI:** 10.7759/cureus.36035

**Published:** 2023-03-12

**Authors:** Ipek Evruke, Inci Sema Tas

**Affiliations:** 1 Obstetrics and Gynecology, Akcakoca State Hospital, Duzce, TUR; 2 Obstetrics and Gynecology, Istanbul University School of Medicine, Istanbul, TUR

**Keywords:** fsfi, operative hysteroscopy, lng-ius, abnormal uterine bleeding, female sexual dysfunction

## Abstract

Background

This study aims to evaluate the effect of operative hysteroscopy and levonorgestrel-releasing intrauterine system (LNG-IUS) insertion for abnormal uterine bleeding on sexual function and, if it improves sexual function, to examine differences in sexual function between women undergoing hysteroscopy and women using LNG-IUS.

Methods

Ninety women aged between 25 and 52 enrolled in the study. Participants were divided into two groups, including 45 operative hysteroscopy patients and 45 LNG-IUS patients. All patients completed the Female Sexual Function Index (FSFI) questionnaire pre-treatment and after three months post-treatment. Pre- and post-treatment FSFI scores were compared both within and between groups.

Results

The mean FSFI scores at three months following both treatments were significantly higher than baseline in both groups (p<0.05). When the two groups were compared, no significant difference was observed between baseline and three-month post-treatment differences in FSFI scores except for the pain domain. In the pain domain of the FSFI questionnaire, a more significant improvement was found in the LNG - IUS inserted group compared to the operative hysteroscopy group.

Conclusion

Patients had improvement in sexual functions after both operative hysteroscopy and LNG - IUS insertion treatment. No significant difference was observed in hysteroscopy and LNG-IUS patients after both treatments in terms of sexual function according to scores calculated by FSFI, except for the pain domain. Significant improvement was observed in the pain domain of the FSFI for the LNG - IUS inserted group compared to the operative hysteroscopy group, thus demonstrating a significant effect and improvement for dyspareunia and chronic pelvic pain complaint in the LNG-IUS inserted group.

## Introduction

Abnormal uterine bleeding (AUB) is a common and significant entity affecting up to 30% of women of reproductive age and up to 50% of premenopausal women [1]. It is characterized by abnormal duration, volume, and/or frequency of uterine bleeding. Women with AUB experience reduced quality of life and may incur high medical costs. The Menstrual Disorders Working Group of the International Federation of Gynecology and Obstetrics (FIGO) has categorized the various underlying conditions that cause AUB and recommended using the “PALM-COEIN’ classification as the systematic terminology for defining AUB [2].

PALM (polyp, adenomyosis, leiomyoma, malignancy/hyperplasia) are structural pathologies that can be measured by using imaging techniques. Non-structural pathologies that cannot be defined by imaging are COEIN (coagulopathy, ovulatory dysfunction, endometrial, iatrogenic, and not yet classified). Treatment options are predominantly surgical in the PALM group and medical in the COEIN group [2]. Structural causes of AUB include endometrial polyps, which are epithelial proliferations arising from the endometrial stroma and glands and can cause intermittent, irregular, heavy menstrual, and postmenopausal bleeding. Leiomyomas are found in almost 50% of women over 35, and submucous leiomyomas are the most likely to cause AUB. Unopposed estrogen has been associated with various endometrial abnormalities ranging from cystic hyperplasia to adenomatous hyperplasia, atypical hyperplasia to invasive carcinoma. These disturbances may have a significant negative impact on the social, physical, and sexual functions and overall quality of life of the patients.

One area of particular concern is the potential impact of heavy menstrual bleeding on female sexual function. Female Sexual Dysfunction (FSD) is defined as a lack of one or more of the components in the sexual response cycle, which includes sexual desire, impaired arousal, and inability to achieve an orgasm or pain with intercourse [3]. Studies have shown that women with AUB may experience various sexual difficulties, including decreased sexual desire, pain during intercourse, and reduced sexual satisfaction. This may be due to various factors, including physical discomfort and anxiety related to bleeding, hormonal imbalances, and psychological distress [4].

Treatment options for AUB depend on the underlying cause and the severity of the symptoms. Various effective medical treatments are available to manage this condition, including NSAIDs, anti-fibrinolytic medications, progestogen-based oral tablets, contraceptive pills, and progestogen-releasing intrauterine systems as surgical procedures such as hysterectomy, hysteroscopic procedures, and endometrial ablation [5].

The levonorgestrel-releasing intrauterine system (LNG-IUS) is a T-shaped device that releases a potent progestin called levonorgestrel (LNG) directly into the uterus at a rate of 20 µg/day over a five- to seven-year period, resulting in a significant decrease in menstrual blood loss. Additionally, it has the potential to alleviate dysmenorrhea and decrease the likelihood of pelvic inflammatory disease by thickening the mucus in the utero-cervical area [6]. Additionally, its reversible removal has minimal impact on long-term pregnancy rates and is a cost-effective and long-term solution that is safe, dependable, and well-tolerated.

Hysteroscopy is considered the gold standard technique for identifying and treating uterine cavity conditions. AUB is the most common indication to perform hysteroscopy in perimenopausal women. The hysteroscopic “see-and-treat” method enables examination of the uterine cavity, targeted biopsies of the endometrial areas, and immediate treatment of endometrial or submucosal pathologies (such as polyps and myomas) if necessary [4]. Minimally invasive operative hysteroscopy treatments have been shown to be effective in treating AUB, improving quality of life, and possibly avoiding or delaying hysterectomy. Removing the polyps is the preferred treatment for treating endometrial polyps that cause AUB symptoms. Hysteroscopic polypectomy is considered the best technique for this purpose and is widely regarded as the most effective way to remove these polyps [7].

The objective of our study was to investigate the variations in sexual function among women experiencing AUB symptoms who underwent operative hysteroscopy and LNG-IUS insertion. Women struggling with heavy menstrual bleeding may experience a considerable decrease in blood loss and comparable quality of life after receiving treatment with either the LNG-IUS insertion or operative hysteroscopy. Limited information is available on the impact of LNG-IUS and operative hysteroscopy treatments on sexual functions independently. Thus, our study aimed to evaluate the effects of both treatments and compare their impacts to contribute to the existing literature by determining which treatment method improves sexual function more effectively.

## Materials and methods

This study was conducted on women who presented to the Akçakoca State Hospital Gynecology Clinic with AUB and were planned to undergo operative hysteroscopy or LNG-IUS insertion after the examination. The study is conducted according to the Good Clinical Practices Guideline of Helsinki. And the Institutional Medical Ethics Committee of Duzce University, Duzce Faculty of Medicine (Protocol number: 2022/179) approved the study procedures. All participants provided written informed consent.

Study design and patient selection

Between May and September 2022, 90 sexually active women aged 25-52 presented with AUB and were planned to undergo a hysteroscopy operation or LNG-IUS insertion were enrolled in the study prospectively. Patients with abnormal bleeding, normal cervical smear results, no history of malignancy, and no known concomitant diseases were included in the study. Subjects were excluded from participation if they had abnormal endometrial biopsy results (malignancy), gynecologic pathologies (endometriosis, myoma uteri, uterine anomalies), and taking medications affecting sexual function. Patients with concomitant diseases (hypertension, diabetes) and grade two or above pelvic organ prolapse were excluded from the study due to their confounding effects on sexual functions. All participants agreed to involve and provided written informed consent for inclusion in the study. 

Patient evaluation

A detailed anamnesis, medical history, menstrual bleeding pattern, lifestyle factors, and concomitant diseases were evaluated. Patients underwent physical and gynecological examinations, a transvaginal ultrasound, and saline contrast sonohysterography (SCSH). The transvaginal probe measured the thickest section of the endometrial layer in the anteroposterior direction. Subsequently, a speculum was inserted into the vagina, and isotonic saline was injected into the uterus via a feeding tube attached to a 20-mL syringe. The uterine cavities were evaluated in both longitudinal and transverse planes. The examinations were conducted on women between the seventh and 10th day following the first day of menstruation. Operative hysteroscopy was performed in cases with suspected focal intrauterine pathology. An endometrial biopsy was performed in cases with irregular/thick endometrium. 

Patients with irregular and increased endometrial thickness were planned to undergo endometrial sampling by endometrial biopsy, followed by LNG-IUS implantation in patients with benign (secretory/proliferative/dysfunctional/atrophic endometrium) results. Endometrial sampling was performed by pipelle endometrial suction curette. Samples were obtained prior to LNG-IUS insertion. Operative hysteroscopy using a resectoscope was performed for patients that were suspected of having endometrial polyps for the cause of AUB by pelvic ultrasound and SCSH. The same specialist performed all procedures. The treatment decision was achieved following the initial evaluation and discussed with the patient. 

The Female Sexual Function Index (FSFI) was used to assess sexual function in both the hysteroscopy group and LNG-IUS insertion group at pre-treatment and three months post-treatment. One patient in the operative hysteroscopy group had a pathology report of an endometrial polyp with endometrial hyperplasia. One patient from the LNG-IUS insertion group had a FIGO-grade 1 endometrial adenocarcinoma, and another one had endometrial hyperplasia in their pathology reports. Therefore, three patients were excluded from the study. All patients were asked to complete the Turkish version of the FSFI to assess sexual function before and at three and six months after treatment to compare the pre- and post-treatment differences.

FSFI assessment

The FSFI is a self-report questionnaire that assesses various aspects of female sexual function [8]. The questionnaire consists of 19 items and measures six domains of sexual function: desire, arousal, lubrication, orgasm, satisfaction, and pain. It uses a scoring system to determine scores in each domain, with higher scores indicating better sexual function. Desire refers to a woman's level of sexual interest and desire. Arousal refers to physiological and psychological responses to sexual stimuli, including lubrication and vaginal swelling. Lubrication refers to the production of natural lubrication in response to sexual stimulation. Orgasm refers to the peak of sexual pleasure and the release of sexual tension. Satisfaction refers to a woman's overall level of satisfaction with her sexual experiences. Pain refers to any pain experienced during sexual activity. Each item in the FSFI is scored on a scale of 0 to 6, and the scores from each of the six domains are combined to form a total score, with higher total scores indicating better sexual function. Women with a score lower than 26.55 were considered to have sexual dysfunction. The FSFI has been validated for use in different cultures and populations and has been shown to have high reliability and validity. A Turkish version of the FSFI, consisting of 19 questions, was created as a concise and comprehensive self-assessment tool to evaluate key aspects of female sexual function [9]. 

Statistical analysis 

Data were analyzed using NCSS (Number Cruncher Statistical System) 2007 (Kaysville, Utah, USA). Power analysis was used, and a total of 90 patients eligible for the study protocol were selected to obtain clinically and statistically significant differences at a 5% significance level, 88% power, an effect size of 0.3, and a correlation between measurements of 0.5. The statistical analysis was done using the paired sample t-test and independent samples t-test. Data were presented as mean ± standard deviation. A p-value of less than 0.05 was considered statistically significant.

## Results

There was a total of 90 patients enrolled in the study. Forty-five of them were planned to have an operative hysteroscopy and 45 of them were planned to have an LNG-IUS insertion. One patient from the operative hysteroscopy group was withdrawn from the study due to a pathology report of an endometrial polyp with endometrial hyperplasia after the operation. Two patients were withdrawn from the LNG-IUS group. One was due to a pathology report of endometrial hyperplasia, and the other was due to a pathology report of FIGO Grade 1 endometrial adenocarcinoma. After excluding these participants, 87 participants, 44 operative hysteroscopy and 43 LNG-IUS insertion groups, were included in the study.

The LNG-IUS insertion and operative hysteroscopy groups had similar mean age, body mass index (BMI), parity, and smoking history. The average age in the operative hysteroscopy group was 41.4 (±7.17) and 39.8 (±6.37) in the LNG-IUS insertion group.

FSFI scores of groups are also shown in Table 1. The prevalence of sexual dysfunction at baseline was 81.3% among LNG-IUS and 81.8% among the hysteroscopy group. The main baseline FSFI scores were found to be similar in both groups. The operative hysteroscopy group had a lower total FSFI score compared to the LNG-IUS group, but the difference was not statistically important. There was no significant difference in the mean scores of all domains of FSFI in both groups.

**Table 1:** Demographic and clinical characteristics of patients among groups.

	Operative hysteroscopy (n=44)			LND-IUS (n=43)			P-value
Age	41.4 ± 7.17			39.8±6.37			0.297
BMI (kg/m^2^)	29.49±4.56			27.95±3.86			0.094
Parity	2.11±1.16			2.13±0.94			0.909
Smoking (n)							0.907
Smoker	20 (45.5%)			19 (44.2%)			
Non-smoker	24 (54.5%)			24 (55.8%)			
FSFI subgroups	Mean±SD			Mean±SD			
Desire	2.97±0.81			3.12±0.93			0.419
Arousal	3.32±0.81			3.32±0.79			0.966
Lubrication	3.83±0.74			3.94±0.66			0.468
Orgasm	3.67±0.80			3.91±0.82			0.165
Satisfaction	3.70±0.84			3.76±0.89			0.718
Pain	4.18±1.04			3.77±1.00			0.068
Total score	21.68±3.11			21.86±3.19			0.795

The scores and the comparison of FSFI scores before and after the hysteroscopy operation are shown in Table 2. The total FSFI scores, median values ± standard deviation, at baseline were 21.68±3.11 and 25.49±2.20 at three months post-treatment follow-up. Pre and post-treatment were for desire 2.97±0.81 and 3.79±0.56, respectively; for arousal 3.32±0.81 and 4.05±0.67, respectively; for lubrication 3.83±0.74 and 4.35±0.58, respectively; for orgasm 3.67±0.80 and 4.20±0.76, respectively; for satisfaction 3.70±0.84 and 4.35±0.61, respectively; for pain 4.18±1.04 and 4.74±0.88, respectively (p<0.01).

**Table 2:** Operative hysteroscopy treatment effect on FSFI scores at pre-treatment and three-month post-treatment follow-up.

FSFI domains	Pre-treatment, Mean ± SD (n=44)	3-Month Follow-up, Mean ± SD (n=44)	P-value
Desire	2.97±0.81	3.79±0.56	<0.01
Arousal	3.32±0.81	4.05±0.67	<0.01
Lubrication	3.83±0.74	4.35±0.58	<0.01
Orgasm	3.67±0.80	4.20±0.76	<0.01
Satisfaction	3.70±0.84	4.35±0.61	<0.01
Pain	4.18±1.04	4.74±0.88	<0.01
Total FSFI score	21.68±3.11	25.49±2.20	<0.01

The total FSFI score before the hysteroscopy operation was found significantly lower than the total FSFI score after the operation (p<0.01). In addition, the scores in all the questionnaire domains were found significantly higher following the operative hysteroscopy.

The scores and the comparison of FSFI scores before and after LNG-IUS insertion are shown in Table 3. The total FSFI scores, median values ± standard deviation, at baseline were 21.86±3.19 and 26.41±2.60 at three months post-treatment follow-up. Pre and post-treatment were for desire 3.12±0.93 and 3.80±0.62, respectively; for arousal 3.32±0.79 and 4.13±0.60, respectively; for lubrication 3.94±0.66 and 4.59±0.63, respectively; for orgasm 3.91±0.82 and 4.53±0.82, respectively; for satisfaction 3.76±0.89 and 4.66±0.65, respectively; for pain 3.77±1.00 and 4.66±0.78, respectively (p<0.01). The total FSFI score before the insertion was considerably lower than the total FSFI score after insertion, with a significance level of p<0.01. In addition, the scores in all the questionnaire domains were significantly higher following the LNG-IUS insertion.

**Table 3:** LNG-IUS treatment effect on FSFI scores at pre-treatment and three-month post-treatment follow-up.

FSFI domains	Pre-treatment, Mean ± SD (n=43)	3-Month Follow-up, Mean ± SD (n=43)	P-value
Desire	3.12±0.93	3.80 ±0.62	<0.01
Arousal	3.32±0.79	4.13 ±0.60	<0.01
Lubrication	3.94±0.66	4.59 ±0.63	<0.01
Orgasm	3.91±0.82	4.53 ±0.82	<0.01
Satisfaction	3.76±0.89	4.66 ±0.65	<0.01
Pain	3.77±1.00	4.66 ±0.78	<0.01
Total FSFI score	21.86±3.19	26.41 ±2.60	<0.01

Table 4 demonstrate the effect of the treatments on sexual function according to operative hysteroscopy/LNG-IUS insertion state at pre-treatment and three months post-treatment. All domains of FSFI were improved in both groups. The improvement in FSFI scores, except for the pain domain, with both treatments, does not significantly differ between groups. The pain domain of FSFI was found to be significantly improved in the LNG-IUS insertion group compared to the operative hysteroscopy group. (p<0.05).

**Table 4:** Improvement in FSFI scores at baseline and three-month follow-up in patients treated with operative hysteroscopy and LNG-IUS insertion.

		Operative hysteroscopy, (n=44)		LND-IUS, (n=43)	P-value
FSFI	Mean	Sd.	Mean	Sd.	
Desire	0.8182	0.6218	0.6837	0.6999	0.346
Arousal	0.7364	0.5030	0.8093	0.6498	0.559
Lubrication	0.5114	0.6423	0.6488	0.6173	0.312
Orgasm	0.5273	0.5087	0.6140	0.5878	0.464
Satisfaction	0.6545	0.5036	0.8930	0.6344	0.055
Pain	0.5636	0.6810	0.8930	0.7992	0.041
Total score	3.8068	1.5512	4.5488	2.5464	0.104

## Discussion

“Sexual health” can be described as a condition that encompasses an individual's overall physical, emotional, mental, and social well-being concerning their sexuality. Maintaining sexual health also involves having a positive attitude towards sexuality and sexual relationships. The etiology of FSD can be multifactorial and can also be affected by various surgical or medical conditions. The biopsychosocial perspective acknowledges that multiple aspects, including biological, psychological, interpersonal, and sociocultural elements, can impact a woman's sexual functioning. These factors interplay within a complex system over time. These biological factors can be hormonal changes that influence desire or medical/anatomical issues that impact genital sexual response. Psychological factors could be symptoms of mood, such as depression or anxiety, or negative attitudes, like being overly self-critical during sexual activity [10]. Physical and mental well-being, illness, and treatments could impact women's desire and sexual responsiveness.

AUB can have a significant impact on sexual function and intimacy, resulting in a decrease in sexual pleasure. Mercer et al. showed that only 21% of women with persistent sexual problems discuss it with their healthcare provider [11]. This issue is often overlooked because patients may feel embarrassed or restricted by social norms, particularly in conservative societies like ours. The condition can cause feelings of anxiety and uncertainty, which can further affect sexual function and intimacy. It becomes challenging to unwind and relish sexual activity, decreasing sexual satisfaction. This situation can lead to a reduction in the frequency and quality of sexual activity, ultimately causing a decrease in the overall health and happiness of the relationship.

Sexual dysfunction can manifest as a range of difficulties that impact sexual desire, arousal, orgasm, and pain during intercourse. These difficulties can lead to significant distress and negatively impact relationships and overall quality of life. Menopause, fatigue and stress, psychiatric and neurologic disease, pelvic floor or bladder dysfunction, endometriosis, uterine fibroids, hypertension, medication and substances, and hormonal contraceptives are known risk factors for FSD [12]. Patients having any of these risk factors were excluded from our study.

The prevalence of FSD is very high in our population. Embarrassment, an essential part of most conservative societies such as ours, may be an important factor for the high rate of FSD. While the FSD rate is reported to be approximately 43%-57% in Turkey, these rates are given as 34%-40% for women in the USA and Europe [13]. The prevalence of FSD among all participants in our study was 81.6% which reduced to 43.6% after both treatments (71/87, 38/87, respectively). The prevalence of FSD in two groups was examined in our study as well. When evaluated individually, the hysteroscopy group had an 81.8% prevalence of FSD prior to treatment, which reduced to 40.9% after treatment (36/44, 18/44, respectively). Similarly, the LNG-IUS group had a prevalence of 81.3% before treatment, which decreased to 46.5% after treatment (36/44, 20/44, respectively). The reason behind the high occurrence of FSD prior to treatment was linked to the fact that the group of patients involved in the study consisted of women with AUB.

Regarding AUB, the quality of life is negatively impacted by various factors such as the unpleasant odor associated with AUB, frequent fungal infections, the irregularity of AUB, and reduced work capacity. Additionally, AUB can result in difficulty with sexual relations. The fear of bleeding or spotting during sexual intercourse can cause embarrassment and avoidance of intercourse. The bleeding can also lead to decreased libido, lubrication, and fear of disappointing a sexual partner. Sexual activity and self-esteem are crucial in affecting the quality of life for individuals with AUB.

The goal of our study was to compare the impact of operative hysteroscopy versus LNG-IUS insertion treatment on the sexual functions of women with AUB symptoms. Our primary outcome was an overall improvement in the sexual functions of all women treated with both treatment methods. We also were interested in the impact of these treatments on specific domains of sexual function, including desire, arousal, lubrication, orgasm, satisfaction, and pain. 

One of the most effective methods for assessing FSD is using verified surveys and symptom scores. We used the FSFI questionnaire to evaluate the patients' sexual functions. The FSFI is widely recognized and preferred for evaluating female sexual functioning. FSFI consists of 19 questions in six domains (desire, arousal, lubrication, orgasm, satisfaction, and pain). Each domain is scored with a minimum and maximum value, and a total score for the sexual function is calculated from all domains. The Turkish version of FSFI has been established to be valid and reliable for evaluating FSD among the Turkish population.

The findings in our study suggest that LNG-IUS insertion improves sexual function in women with AUB. Skrzypulec et al. conducted a study on a total of 200 women divided into three groups: women using LNG-IUDs, women using other IUDs, and women using no IUDs [14]. LNG-IUS inserted group had shown a significantly better quality of life and sexual functions compared to other groups. Women in our study showed marked improvements in their FSFI scores after the LNG-IUS insertion. Additionally, all domains of the FSFI questionnaire revealed statistically significant improvements post-insertion. In a retrospective study by Gorgen et al. on LNG-IUS treatment for AUB, the scores for pelvic pain measured on a visual analog scale (VAS) decreased from baseline to six-month follow-up [15]. Additionally, the libido and overall health scores as measured on the VAS improved, increasing from 4.3 to 5.0 and 3.5 to 6.9, respectively. 

Neri et al. conducted a study where 31 individuals inserted LNG-IUSs [16]. After a year of monitoring, the researchers found no impact of LNG-IUS on sexual function. However, our study saw significant enhancements in sexual function evaluations. The disparity in the results could be due to the differing LNG doses or the study group difference. The previous study was conducted on healthy premenopausal women and used a low dose of LNG (6 mcg/day), while we utilized a higher dose of LNG (20 mcg/day) on women with AUB complaints. It has been reported that LNG-IUSs worsen sexual function in Ferreira et al.'s study [17]. These results are mainly due to the unplanned spotting effect. However, the inclusion criteria and characteristics of the patient population were not the same as our study. Since our study was based on women with AUB complaints, sexual function improvement was predicted with decreased bleeding symptoms regardless of spotting complaints.

Less is known about the impact of operative hysteroscopy procedures on sexual functions. A review of the existing literature reveals no clinical studies assessing the effect of hysteroscopic polypectomy on sexual functions and only a few studies comparing the effects of medical and surgical treatments of AUB on sexual functions. Only a few studies on small groups of women who underwent hysteroscopic endometrial ablation have shown improved well-being and sexual function.

Marnach et al. showed that undergoing hysteroscopic endometrial ablation for AUB resulted in an improvement in female sexual function and a decrease in personal distress [18]. On the other hand, Zhang et al. compared the effects of laparoscopic and hysteroscopic myomectomy on sexual desire, sexual arousal, vaginal lubrication, orgasm, sexual satisfaction, and sexual intercourse pain in women. They did not find any changes in sexual functions before or at the three and six-month follow-ups after surgery.

Our study observed significant enhancements in FSFI scores for women following the hysteroscopy procedure. Moreover, all domains of the FSFI survey demonstrated notable and statistically significant enhancements after the operation. The performed operative hysteroscopy reduced the symptoms associated with sexual intercourse and thus improved the quality of life. It is essential to emphasize that the relief of symptoms was declared after operative hysteroscopy.

There were no significant differences between the two groups in terms of pre and post-operative sexual desire, sexual arousal, vaginal lubrication, orgasm, and sexual satisfaction (p>0.05). Whereas the improvement in FSFI scores was significant overall in both groups, the pain domain of the FSFI survey showed a statistically significant improvement in the LNG-IUS inserted group compared to the group who had hysteroscopy (p<0.05).

Chronic pelvic pain is challenging to manage, and treatment options vary depending on the underlying cause. The LNG-IUS is a safe and effective treatment option for many women with chronic pelvic pain. Studies have demonstrated that the LNG-IUS can lower the amount of blood flow in both the uterine artery and the subendometrial spiral arteries. This decrease in blood flow could be a possible reason for the relief of pelvic pain in women with the LNG-IUS inserted [19]. Therefore, the improvement in the pain domain in the LNG-IUS inserted group is consistent with the literature. 

A study conducted by Radzinsky et al. showed that women with chronic pelvic pain who had an LNG-IUS inserted experienced a significant improvement in their quality of life, and the pain scores had improved significantly compared with baselines at 12 months of follow-up after insertion [20].

The strengths of our present study include the prospective enrollment of the patients and the usage of a validated survey instrument to assess the sexual function of the patients. A relatively small sample size, and single-center experience, are the significant limitations of our study.

## Conclusions

Both LNG-IUS insertion and operative hysteroscopy have been shown to be effective options for women with AUB who are concerned about their sexual well-being and want to avoid a hysterectomy. Using a validated survey instrument (FSFI) to assess sexual function is also a positive development in understanding the impact of these treatments on overall well-being.

It is essential to continue researching and studying the effects of medical and surgical treatments on women's physical, emotional, and sexual well-being to ensure that the best possible outcomes are achieved and to improve the quality of life for women with AUB. By understanding the impact of these treatments on all aspects of a woman's life, healthcare providers can offer more personalized and effective treatment options.
